# Determinants of Adoption of a Mechanical Ventilation Dashboard in Intensive Care: Measurement Instrument for Determinants of Innovations–Based, Single-Center, Cross-Sectional Evaluation

**DOI:** 10.2196/92313

**Published:** 2026-10-07

**Authors:** Sarah Frederika Christina Mugge, Tobias Daniel van Leijsen, Merijn Constantijn Reuland, Annelies Visser, Frederique Paulus, Dave Anton Dongelmans

**Affiliations:** 1 Department of Intensive Care, Amsterdam University Medical Centers, Meibergdreef 9, Amsterdam, North Holland, 1105 AZ, The Netherlands, 31 20-566 9111; 2 Department of Surgery, Amsterdam University Medical Centers, Amsterdam, North Holland, The Netherlands; 3 Faculty of Health, Center of Expertise Urban Vitality, Amsterdam University of Applied Sciences, Amsterdam, North Holland, The Netherlands; 4 Amsterdam Public Health Research Institute, Amsterdam University Medical Centers, Amsterdam, North Holland, The Netherlands

**Keywords:** adoption, barriers and facilitators, dashboard, implementation, intensive care unit, mechanical ventilation, Measurement Instrument for Determinants of Innovations, MIDI questionnaire, lung-protective ventilation, ventilation dashboard

## Abstract

**Background:**

Lung-protective ventilation (LPV) reduces complications of mechanical ventilation, yet adherence in intensive care units (ICUs) remains inconsistent. Digital dashboards may support LPV by improving situational awareness and supporting protocol adherence. However, adoption of such tools in high-acuity clinical environments depends on a range of cognitive, professional, and contextual determinants. The Measurement Instrument for Determinants of Innovations (MIDI) provides a validated framework to systematically assess these factors.

**Objective:**

This study aims to identify determinants influencing the adoption of a newly piloted mechanical ventilation dashboard in the ICU using the MIDI framework.

**Methods:**

We conducted a single-center, cross-sectional evaluation among ICU health care professionals during a dedicated survey period within a pilot introduction of a mechanical ventilation dashboard at Amsterdam University Medical Center. Participants completed a structured questionnaire consisting of 24 MIDI items adapted to the ICU context rated on a 5-point Likert scale (“completely disagree” to “completely agree”), supplemented by open-ended questions on perceived barriers and facilitators to its use. Determinants were classified as facilitators when ≥80% of respondents selected “agree” or “completely agree” and as barriers when ≥20% selected “disagree” or “completely disagree.” Open-ended responses were analyzed using a general inductive thematic approach.

**Results:**

A total of 71 completed questionnaires were analyzed, including responses from nurses, physicians, intensivists, ventilation specialists, and researchers in mechanical ventilation. Six determinants met the criteria for facilitators: outcome expectations, self-efficacy, procedural clarity, low complexity, correctness, and observability. Two determinants met the criteria for barriers: relevance for client and professional obligation. Analysis of open-ended responses highlighted perceived barriers such as additional workload, the need for an extra device, overlap with existing systems, and limited role-specific relevance. Facilitators included improved situational overview, educational value, easier trend monitoring, and increased efficiency.

**Conclusions:**

This evaluation identified key determinants influencing the adoption of a mechanical ventilation dashboard in the ICU. While the dashboard was generally perceived as useful and easy to understand, adoption was shaped by determinants related to workflow integration, role-specific relevance, and professional responsibility. These findings suggest that successful introduction of digital clinical support tools in intensive care requires attention not only to technical design, but also to how such tools align with users’ roles, daily work processes, and shared clinical responsibilities. Systematic assessment of determinants provides actionable insights into the adoption of digital decision-support tools in high-acuity care settings.

## Introduction

Mechanical ventilation is a critical and often lifesaving intervention for patients admitted to intensive care units (ICUs). However, mechanical ventilation can also contribute to lung injury and complications, including acute respiratory distress syndrome and ventilator-induced lung injury, which increase morbidity and mortality [1,2]. To reduce these risks, clinicians use lung-protective ventilation (LPV) strategies, including low tidal volume (4‐8 milliliters/kilogram ideal body weight [V_T_/IBW]), limited plateau pressures (<30 cm H₂O), and limited driving pressures (<15 cm H₂O) [2-6].

Despite strong evidence, adherence to LPV in daily ICU practice remains inconsistent [7,8]. Both patient factors, such as body size and disease severity, and organizational factors such as protocols, staffing and unit routines, contribute to variation in LPV delivery [8-10]. In addition, high cognitive workload, competing clinical demands, and limited protocol visibility can hinder consistent application of evidence-based ventilation practices [11,12].

Clinical dashboards may support LPV by integrating relevant data into a single view, improving situational awareness, and reducing cognitive effort during decision-making [13,14]. In the context of LPV, a ventilator management dashboard with tidal volume alerts was associated with improved hospital-wide adherence and facilitated timely correction of suboptimal ventilator settings [15,16]. Beyond adherence, dashboards may also increase efficiency in prerounding data gathering and, when displayed on shared screens, enhance communication accuracy, information exchange, and clinical satisfaction during multidisciplinary discussions [17]. More broadly, prior studies have shown that clinical dashboards can support care delivery by integrating relevant data, reducing cognitive burden, and providing real-time feedback, which may contribute to improved guideline adherence and fewer errors in complex clinical settings [13,14,18].

Adoption of change in the ICU is shaped by perceived role relevance and feasibility within existing workflows, and influenced by a complex and multifaceted set of facilitators and barriers at the personal, organizational, and health system levels, including engagement, available resources, and local organizational context [19-23]. To systematically assess these factors, the Measurement Instrument for Determinants of Innovations (MIDI) has been widely applied in health care settings [24,25]. The MIDI extends beyond technical considerations by capturing determinants such as procedural clarity, compatibility with daily practice, and relevance for professionals and patients. Previous research has demonstrated its utility in identifying barriers and facilitators that influence the adoption of new technologies and interventions in clinical practice [26-30].

The aim of this study was to use the MIDI questionnaire to identify barriers and facilitators influencing the adoption of a newly piloted mechanical ventilation dashboard designed to enhance LPV in the ICU.

## Methods

### Design, Setting, and Participants

We conducted a single-center, cross-sectional questionnaire study among ICU health care professionals in a Dutch tertiary academic referral center, where a mechanical ventilation dashboard was introduced as part of a pilot implementation. The study had a descriptive and exploratory aim: to identify determinants influencing adoption of the dashboard in the ICU context. The study took place at Amsterdam University Medical Center (Amsterdam UMC).

The study population consisted of ICU health care professionals involved in mechanical ventilation, including intensivists, ICU nurses, physicians, ventilation practitioners (ICU nurses with additional training as ventilation practitioners), and researchers in the field of mechanical ventilation.

### Ethical Considerations

The Medical Ethics Review Committee of Amsterdam UMC reviewed the study protocol and confirmed that the Dutch Medical Research Involving Human Subjects Act (WMO) did not apply to this study, and gave approval for this study (reference: 2025.0194). Participation was voluntary and informed consent was implied by completion of the questionnaire. Questionnaire responses were pseudonymized, stored securely, and handled confidentially. No financial or other incentives were provided for participation.

### Innovation: VentSight Ventilation Dashboard

The innovation under study was the VentSight ventilation dashboard, a web-based application developed by Hamilton Medical. The dashboard was installed in the ICU of Amsterdam UMC during the first months of 2025 and made available to authorized staff and researchers as part of routine pilot use. The present study evaluated user perceptions collected during a dedicated MIDI survey period conducted within this pilot.

The dashboard was designed to support evaluation of and adherence to LPV by providing continuously updated visualizations of key ventilation parameters. Deviations from predefined target ranges, derived from the hospital’s local ventilation protocol, were highlighted to facilitate timely recognition and response. Target ranges varied according to the selected clinical protocol (acute respiratory distress syndrome, obstructive lung disease, normal LPV, or venovenous extracorporeal membrane oxygenation). Protocol selection was performed manually to align with the patient’s clinical context. Access to the dashboard for authorized users was provided through a secure environment within the hospital’s information technology domain.

For patients receiving invasive or noninvasive ventilation, the dashboard presented 2 levels of information: a unit-wide overview display and individual ventilator detail pages. The overview displayed all active ventilators on a single screen, including positive end-expiratory pressure (PEEP), fraction of inspired oxygen (FiO₂), V_T_/IBW, ventilation mode, and basic patient characteristics (sex and height). Parameters were updated every minute, and values outside target ranges were highlighted, accompanied by trend arrows indicating changes over the preceding hours.

Selecting an individual ventilator opened detailed modules showing graphical trends such as FiO₂, PEEP, V_T_/IBW, respiratory rate and driving pressure, a weaning module aligned with local protocols, continuously refreshed pressure and flow waveforms, and additional ventilator readouts such as cumulative ventilation duration. An example of the dashboard interface is presented in Figure 1. The dashboard and implementation process are further described using the TIDieR (Template for Intervention Description and Replication) guidelines (Checklist 1).

The dashboard’s design supported use across professional roles. For nurses, reviewing individual ventilator parameters for assigned patients aligned with their day-to-day bedside responsibilities. For physicians and ventilation practitioners, the unit-wide overview aligned with their roles in monitoring ventilation practices across the whole unit and identifying ventilators requiring additional attention. During the pilot period, the dashboard was not integrated into standard clinical care. Access to and use of the dashboard were limited to study participants during the study procedures and were voluntary. Dashboard use took place under the supervision of the leading researcher (SFCM) to prevent unintended clinical use.

**Figure 1.**
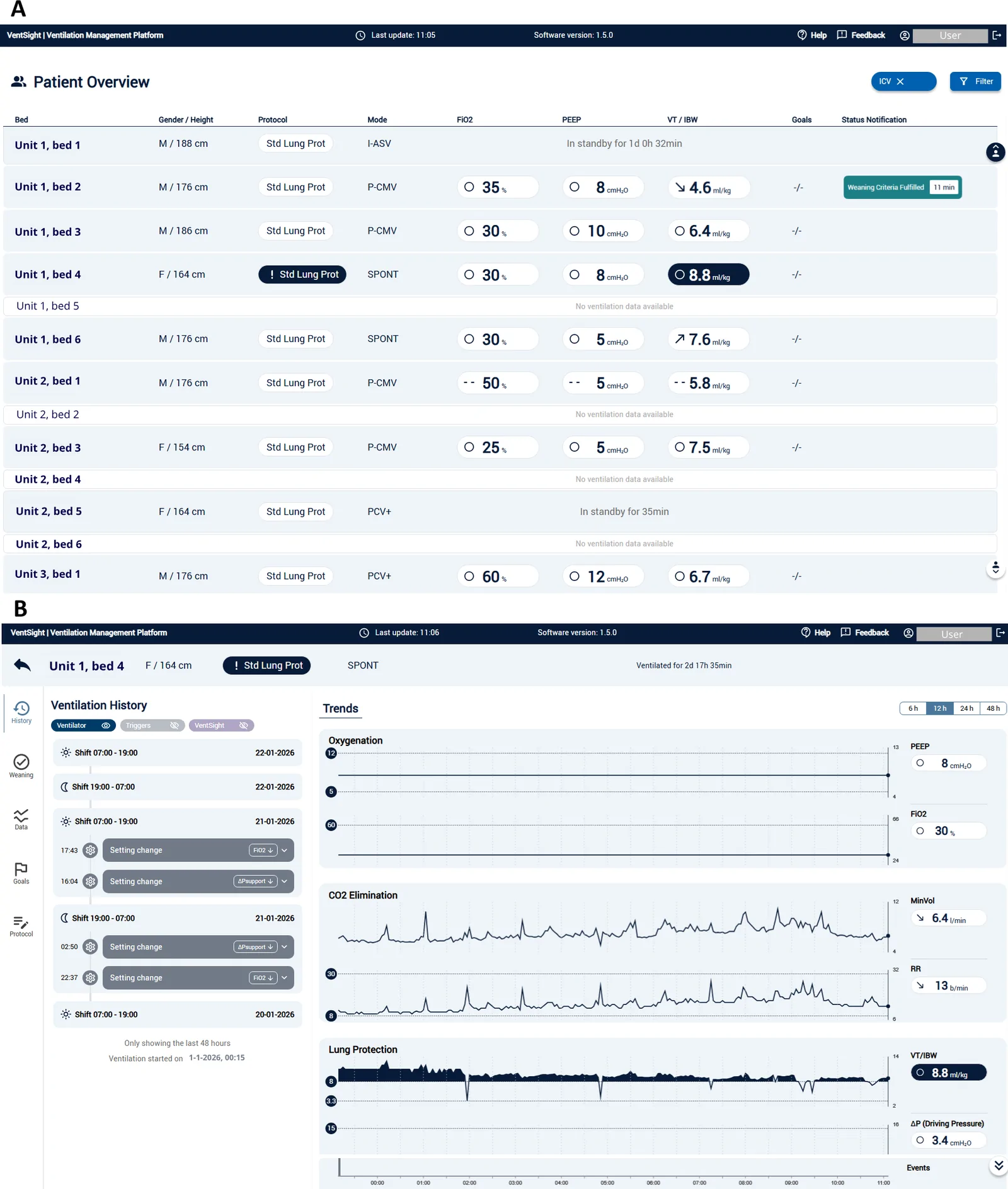
Example of the ventilation dashboard’s interface. The dashboard consists of 2 main layers: (A) the intensive care unit (ICU)–wide overview page, displaying all ventilators, and (B) the individual ventilator page, showing trends, waveforms, and protocol-relevant ventilator parameters.

### MIDI-Based Determinants Assessment

The study intervention consisted of administering the MIDI questionnaire to identify determinants influencing the adoption of the ventilation dashboard in daily ICU practice [25].

### Questionnaire Development

Determinants were assessed using the MIDI framework. The MIDI is intended to be tailored to the specific innovation and implementation context under study. Therefore, in this study, the MIDI was used as intended and tailored to the VentSight dashboard context without redeveloping the questionnaire or changing the underlying MIDI determinant structure. From the original 28 MIDI determinants, 17 were considered relevant to the ICU context and operationalized into 24 questionnaire items rated on a 5-point Likert scale (“completely disagree” to “completely agree”). Items were framed around the use of the dashboard to support LPV, including timely recognition of deviations and adherence to local ventilation protocols.

Several MIDI determinants, such as personal benefits and outcome expectations, were operationalized using multiple items, which are reported separately in the “Results” section. Additional questions captured professional role, intention to use the dashboard (yes, no, or maybe), and open-ended reflections on perceived barriers and facilitators. A complete overview of items is provided in Multimedia Appendix 1.

The questionnaire was developed by a multidisciplinary team, including an implementation specialist, intensivist, ICU nurse, and physician, and was tailored into 2 role-specific versions. ICU nurses received a version aligned with individual ventilator assessment (individual assessment setup), whereas physicians, ventilation practitioners, and researchers received a version emphasizing the unit-wide overview (ICU overview setup). Both versions contained the same 24 MIDI items; only wording and examples were adapted to reflect role-specific use. The questionnaire was built and administered using Castor EDC.

### Pilot Testing

Three ICU ventilation practitioners piloted the questionnaire to evaluate face validity, clarity, and feasibility of completing the questionnaire within the intended time frame (20 min). Feedback indicated that all items were understandable and could be completed within 15 minutes. Minor revisions were made to improve phrasing and logical flow. Pilot testing, therefore, focused on usability and contextual clarity rather than psychometric revalidation of the MIDI instrument itself.

### Questionnaire Administration and Recruitment

Information about the study and the dashboard was disseminated 1 week before and during the questionnaire administration period via posters and the digital ICU newsletter. The MIDI questionnaire was administered between July 28, 2025, and August 4, 2025. The target sample size and distribution across professional groups were determined pragmatically, in line with the descriptive and exploratory purpose of this single-center implementation study. As the study aimed to identify determinants of dashboard adoption in the local ICU context rather than to test a predefined hypothesis or estimate an intervention effect, no formal power-based sample size calculation was performed.

Participants were recruited using a stratified convenience sampling approach. Professional groups involved in mechanical ventilation and dashboard use were considered relevant strata, and recruitment was targeted to ensure participation from each of these groups. Within these strata, participants were recruited based on availability and willingness to participate, rather than through random sampling. We aimed for approximately 75 respondents to obtain a broad representation of intended end users. This included approximately 40 ICU nurses, representing about 20% of the approximately 200 ICU nurses working in the ICU, and approximately half of the available physicians and intensivists involved in ICU ventilation care. The higher target number for ICU nurses reflected their larger share of the ICU workforce and their role as key end users of the individual ventilator assessment view. This approach was considered appropriate for the exploratory implementation-focused aim of the study and feasible within the local pilot implementation period.

All participants received a standardized 5- to 10-minute face-to-face onboarding session led by 1 researcher (SM), ensuring consistent exposure to the dashboard’s key function. Participants then briefly explored the dashboard using short exploratory questions (Multimedia Appendix 2), followed by completion of the pseudonymized questionnaire via a QR code linking to the secure Castor EDC platform. Therefore, responses reflected initial perceptions after short-term, voluntary exposure, rather than experience with sustained use in routine clinical practice. All sessions were facilitated by the same researcher to ensure consistency of exposure. No modifications were made to the dashboard or study procedures during the questionnaire administration period.

### Statistical Analysis

All analyses were descriptive, consistent with the exploratory aim of this single-center implementation study. Questionnaires with less than 70% completion were excluded from analysis to ensure adequate data completeness. For each MIDI item, we calculated the median and IQR, as well as the proportion of respondents selecting “agree” or “strongly agree” (scores 4 or 5) and “disagree” or “strongly disagree” (scores 1 or 2).

In accordance with the MIDI developer recommendations, determinants were classified as facilitators when ≥80% of respondents scored 4 or 5 and as barriers when ≥20% scored 1 or 2 [30]. Determinants were ranked descriptively by these proportions in the combined sample and examined separately by dashboard setup (individual assessment vs ICU overview) to account for differences in use context. Responses to open-ended questions were analyzed using a general inductive approach; responses were read iteratively and grouped into recurring thematic categories to summarize reported determinants. All analyses were performed using R statistical software (version 4.3.3; RStudio).

## Results

### Participant Characteristics

A total of 78 questionnaires were started. After exclusion of questionnaires with less than 70% item completion, 71 questionnaires were included in the analysis: 36 from the individual assessment setup and 35 from the ICU overview setup. Respondents represented a wide range of ICU professional roles, with ICU nurses forming the majority (39/71, 54.9%), followed by physicians (16/71, 22.5%) and intensivists or intensivist in training (11/71, 15.5%). Five respondents were specialized in mechanical ventilation, either as ventilation practitioners or members of their ICU’s ventilation work group. Respondent characteristics are shown in Table 1.

Overall, 51 out of 71 (71.8%) respondents indicated that they intended to use the dashboard, while 20 out of 71 (28.2%) respondents answered “maybe.” No respondents indicated that they did not intend to use the dashboard. In the individual assessment setup, 28 out of 36 (77.8%) respondents indicated an intention to use, compared with 23 out of 35 (65.7%) respondents in the ICU overview setup.

**Table 1.** Respondent characteristics (N=71).

Characteristics	Value
Professional role, n (%)	
ICU^a^ nurse	39 (54.9)
ICU nurse in training	7 (17.9)
ICU nurse (<5 years of experience)	16 (41)
ICU nurse (>5 years of experience)	16 (41)
Physician	16 (22.5)
Intensivist	10 (14.1)
Intensivist in training	1 (1.4)
Researcher in the field of mechanical ventilation	5 (7.0)
Specialized in mechanical ventilation^b^	5 (7.0)
Comfortable with applying mechanical ventilation on a 9-point Likert-scale, median (IQR)	8 (6-8)

a ICU: Intensive care unit.

b Besides other professional role; for example, a nurse who is also a ventilation practitioner or intensivist who is a member of a ventilation working group.

### Facilitators for Implementation

Six determinants met the predefined criteria for classification as facilitator (Figure 2). These included outcome expectations (MIDI item 9a, reflecting the importance of maintaining patients within protocol-defined ventilation targets), self-efficacy (MIDI item 16a, related to recognizing deviations from the ventilation protocol using the dashboard), procedural clarity, low perceived complexity, correctness of the dashboard, and observability. Outcome expectations and self-efficacy received the highest levels of agreement, with 97% (68/70) and 87% (61/70) of respondents, respectively, scoring “agree” or “strongly agree.” Of the 6 determinants classified as facilitators, 2 were related to the end user (outcome expectations and self-efficacy), whereas the remaining 4 facilitators were related to the innovation (procedural clarity, low perceived complexity, correctness, and observability).

**Figure 2.**
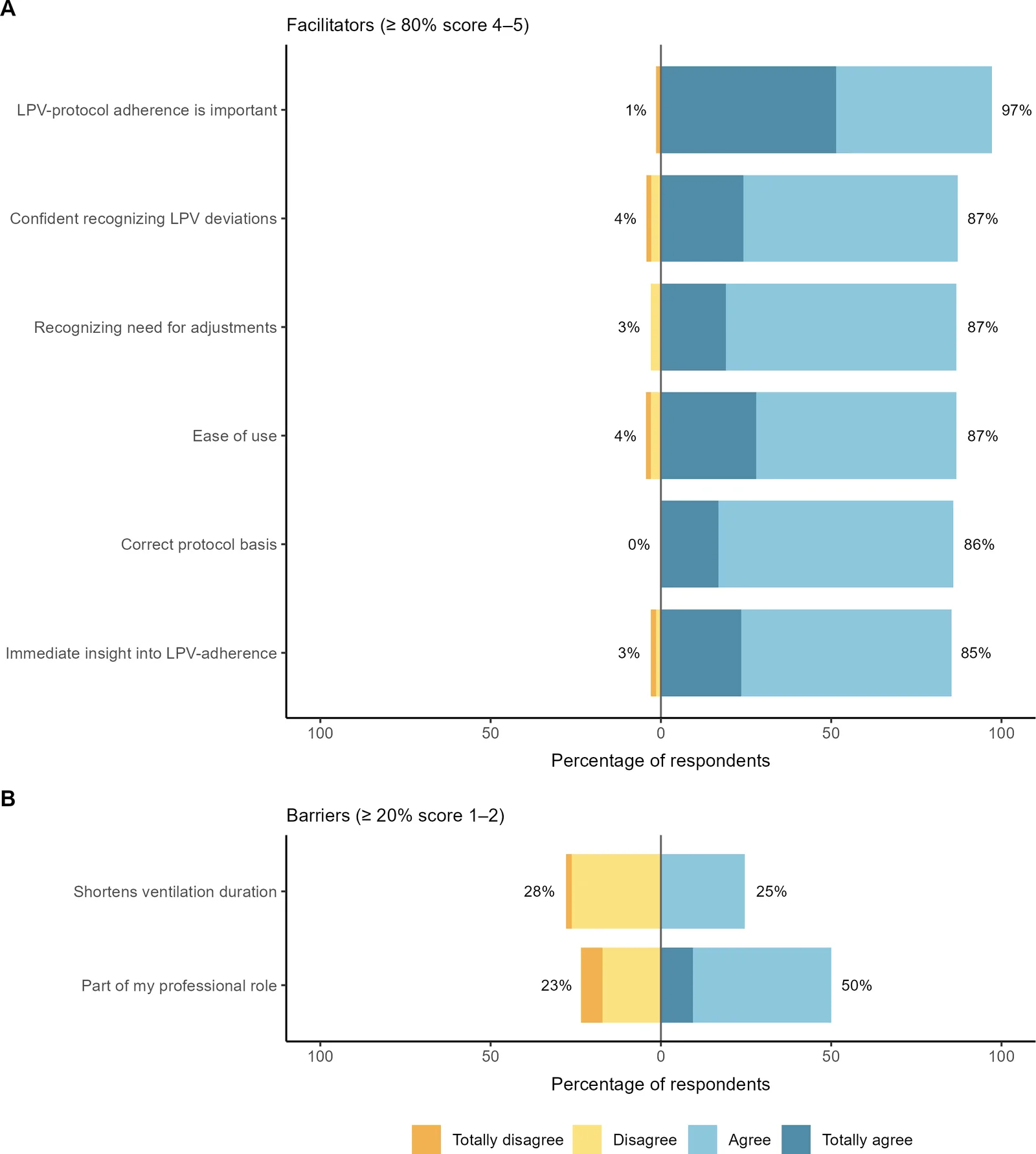
Facilitators and barriers based on Measurement Instrument for Determinants of Innovations (MIDI) cutoffs. (A) Facilitators and (B) barriers identified among all participants based on the predefined MIDI thresholds. Facilitators were defined as MIDI items for which ≥80% of respondents scored 4–5, whereas barriers were defined as MIDI items for which ≥20% of respondents scored 1-2. LPV: lung-protective ventilation.

### Barriers for Implementation

Two determinants met the predefined criteria for classification as barriers (Figure 2). These were relevance for client (MIDI item 7b, reflecting uncertainty about whether dashboard use would shorten mechanical ventilation); and professional obligation. Disagreement was reported by 28% (17/61) of respondents for relevance for client and by 23% (15/64) for professional obligation. Relevance for client is associated with the innovation, whereas professional obligation is associated with the adopting user. An overview of all determinants is presented in Figure 3.

**Figure 3.**
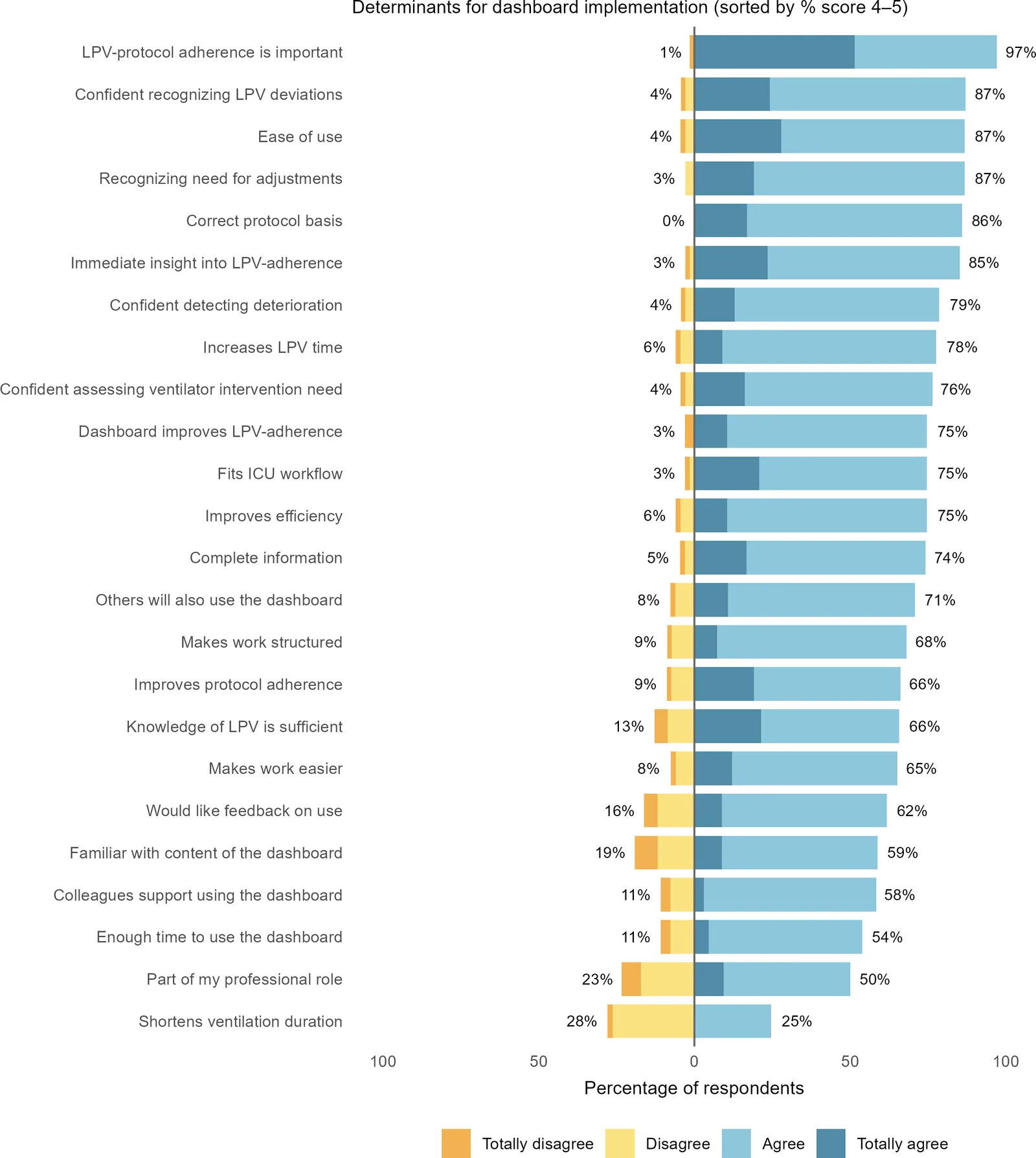
Likert responses for all Measurement Instrument for Determinants of Innovations (MIDI) items sorted by agreement. Likert-scale responses (1-5) for all MIDI items, ordered by the proportion of respondents scoring 4-5 among all participants. ICU: intensive care unit; LPV: lung-protective ventilation.

### Determinants Across Dashboard Setups

Facilitators differed by dashboard use context. Six facilitators were identified in the individual assessment setup, including outcome expectations (MIDI item 9a, importance of maintaining patients within protocol-defined ventilation targets), correctness, observability, self-efficacy related to recognizing protocol deviations and deteriorating ventilator settings, and low perceived complexity. Nine facilitators were identified in the ICU overview setup, including procedural clarity, outcome expectations, multiple self-efficacy items related to protocol monitoring, low perceived complexity, observability, perceived personal benefits related to efficiency, correctness, and compatibility with workflow (Multimedia Appendix 3).

Across both setups, relevance for the client (MIDI item 7b, reflecting uncertainty about whether dashboard use would shorten mechanical ventilation), and professional obligation were consistently identified as barriers. Feedback on performance was an additional barrier in the individual assessment setup, while limited awareness of the innovation’s content emerged as a barrier in the ICU overview setup. Detailed item-level results are provided in Multimedia Appendix 4.

### Perceived Barriers and Facilitators

Responses to open-ended questions revealed that perceived barriers included the dashboard being considered extra work and time-consuming, requiring an additional device, presenting information already available in existing systems, and being perceived as not relevant to certain users’ roles (particularly nurses).

Facilitators included providing the unit with a broader overview of all patients, supporting education, and enabling easier trend monitoring. Reported benefits were improved efficiency and time savings, enhanced situational overview, and the ability to monitor multiple patients simultaneously. Reported disadvantages were the need for an additional screen and the potential for increased screen-focused work, which could reduce direct patient contact. All open-ended responses are presented in Multimedia Appendix 5.

## Discussion

### Main Findings

Using the MIDI, we found that adoption of the dashboard aimed at supporting LPV was mainly facilitated by high perceived outcome expectations, self-efficacy, and procedural clarity, while relevance for the client and professional obligation emerged as barriers to implementation. Facilitators and barriers differed slightly by dashboard use context. Most respondents intended to use the dashboard in clinical practice.

### Strengths

This study has several strengths. The questionnaire was developed using the validated MIDI framework [24,25], ensuring systematic assessment of a wide range of determinants for implementation. The questionnaire was tailored to 2 user contexts (individual assessment- and ICU overview setup) and piloted for clarity and feasibility. Recruitment during active demonstration of the dashboard ensured that respondents were familiar with the tool before providing feedback. The combination of quantitative Likert-scale items with open-ended questions provided both structured and nuanced insights into perceived facilitators and barriers. The MIDI provided a structured approach to assess determinants of dashboard adoption in the ICU context. Combining structured ratings with open-ended responses allowed us to identify facilitators and barriers and to descriptively compare findings across dashboard use contexts.

### Limitations

Some limitations must be considered. First, the study was conducted in a single tertiary ICU in the Netherlands, which may limit generalizability to other settings with different workflows, staffing models, or digital infrastructure. Although the specific determinants identified are context- and innovation-dependent, several reflect broader features of ICU practice and may therefore be relevant beyond this single dashboard. Moreover, this study illustrates how a framework-based approach, such as the MIDI, can be used to systematically identify context-specific barriers and facilitators for digital tool adoption across ICU settings. Second, this was a descriptive and exploratory implementation study with a pragmatically determined sample size. The study was not designed or powered to test predefined hypotheses, estimate intervention effects, or perform formal subgroup comparisons. The findings should therefore be interpreted as context-specific insights into determinants of dashboard adoption, rather than as precise estimates generalizable to all ICU health care professionals. In addition, the professional distribution of respondents was pragmatically determined and was not based on formal stratified random sampling, which may limit interpretation of differences between professional groups. Third, participation was voluntary, which may have introduced selection bias; those more interested in innovation or ventilation practices may have been more likely to respond. Social desirability bias may also have influenced responses, despite pseudonymization, as the dashboard was a local initiative. Fourth, respondents were introduced to the dashboard through a brief standardized onboarding session rather than through prolonged real-world use, and dashboard use was voluntary and limited to study participants. The timing and voluntary context should be considered when interpreting perceived ease of use and intention to use [31]. Initial perceptions shortly after introduction may differ from perceptions after sustained routine use, particularly once users have more opportunity to evaluate workflow integration, peer use, and the dashboard’s practical contribution to ventilation care. To partially mitigate limited exposure, participants were given time to explore the dashboard themselves by completing several short explanatory tasks, allowing hands-on interaction that approximated early real-world exposure. Finally, as this was a perception-based questionnaire, findings do not directly measure actual changes in LPV adherence or patient outcomes.

### Relation With Prior Work

Our findings align with previous studies showing that adoption of change and implementation of digital tools in ICU settings are shaped by a complex and interconnected set of facilitators and barriers operating across individual, organizational, and health system levels, rather than by technological usability alone [20-23]. Consistent with earlier studies, determinants identified in our study emphasize the importance of staff engagement, professional beliefs, and perceived relevance of the innovation, alongside feasibility within existing workflows and workload constraints [20,23]. Both the MIDI-based assessment and the open-ended responses suggest that barriers to adoption predominantly arise from challenges related to integration into daily practice, competing clinical demands, and unclear professional responsibility, reinforcing the notion that successful uptake of digital tools in critical care depends on alignment with human and organizational factors within the ICU context [13-15,19].

### Interpretation of Findings

The high intention-to-use rate suggests initial acceptability of the dashboard among a wide range of ICU professionals, although this should be interpreted in the context of voluntary participation and short-term exposure during a study-specific onboarding session. This finding can be interpreted in light of technology acceptance models such as the technology acceptance model (TAM) and TAM2, in which perceived usefulness and perceived ease of use are important predictors of usage intention and actual system use [31,32]. Although these constructs were not directly measured by the MIDI, several identified facilitators may reflect conditions that support them. High scores for procedural clarity, low perceived complexity, correctness, and observability suggest that the dashboard was experienced as clear, reliable, and straightforward to interpret, supporting perceived ease of use in a complex clinical environment. High outcome expectations and self-efficacy may similarly reflect perceived usefulness and confidence in using the dashboard for protocol monitoring. Together, these findings may help explain the high initial intention to use the dashboard.

However, our findings indicate that successful implementation is unlikely to be achieved by introducing the technology alone; it requires clear integration strategies into daily workflows. This is particularly relevant in light of TAM2, which emphasizes job relevance as an important determinant of perceived usefulness and user acceptance. In our study, low scores for relevance for client, reflecting uncertainty about patient-related benefit, and professional obligation emerged as barriers [31]. This suggests that the dashboard’s relevance for patient care and its place within shared professional responsibilities were not yet fully established across professional groups. The lack of perceived personal benefit, particularly among respondents in the individual assessment setup, may further reduce the likelihood of sustained use. Addressing these barriers should therefore be a key focus of future implementation strategies, including targeted education on the downstream clinical impact of consistent LPV and explicit clarification of how dashboard use contributes to team-based ventilation care.

Because the dashboard was not yet integrated into standard clinical care, respondents’ perceptions may differ from perceptions after sustained routine use. In particular, perceived usefulness may increase once users have more opportunity to experience how the dashboard fits into daily workflow, supports shared situational awareness, and contributes to ventilation monitoring over time. Although continuous display of the dashboard at the nursing station was not formally evaluated in this pilot, our findings suggest that such continuous visibility may further reinforce situational awareness and promote routine use, particularly among bedside nurses. In addition, introducing a structured “ventilation review moment” on each ICU, using the dashboard as a central reference tool, could support its use in daily practice, promote protocol adherence, enhance shared situational awareness, align perspectives on ventilation status across professionals, and reinforce shared responsibility for LPV.

Several determinants, including time available, social support, and awareness of the innovation’s content, did not meet formal barrier thresholds but showed comparatively low scores. These factors may reflect practical constraints and limited peer reinforcement rather than definitive barriers, yet they represent areas of vulnerability that could hinder adoption if left unaddressed. Embedding dashboard use within a dedicated, team-based ventilation review moment may help mitigate these constraints by normalizing use, strengthening shared responsibility, and increasing familiarity with the dashboard’s functionality.

Although the specific configuration and relative importance of determinants identified in this study are shaped by the local ICU context and the characteristics of this dashboard, the findings may point to more general challenges inherent to implementing digital support tools in critical care settings. Determinants such as limited time availability, uncertainty about perceived patient-related benefits, and unclear professional responsibility may reflect underlying features of ICU work, including high workload, task fragmentation, and shared responsibility across professional roles. Rather than implying that these determinants apply uniformly across settings, our findings illustrate how implementation challenges in critical care often arise from the interaction between human, organizational, and contextual factors, extending beyond technical usability alone.

These findings indicate that implementation strategies for nurses should focus on building buy-in and peer support for dashboard use, allocating protected time, and reinforcing the relevance and positive effects of LPV. For physicians, role-specific strategies should emphasize education highlighting the patient benefits of LPV and emphasizing that delivering LPV as a team aligns with professional responsibilities. Practical next steps include tailoring role-specific dashboard views, integrating the dashboard directly into existing systems, and introducing structured moments to jointly assess and evaluate ventilation practices. Finally, clearly communicating the rationale and benefits of LPV, while fostering a culture in which feedback on ventilation practices is normalized and acted upon, may substantially enhance adoption and quality of patient care.

### Conclusions

Using the MIDI questionnaire to evaluate adoption of a ventilation dashboard, we found that ICU professionals generally perceived the dashboard as feasible and useful and reported a high intention to use it, while also identifying adoption barriers. Our findings highlight that successful adoption of digital dashboards in critical care depends on human, organizational, and contextual factors at the workplace. Recognizing and addressing these factors is essential for implementing digital tools that aim to improve quality of care in the ICU.

## Supplementary material

10.2196/92313 Multimedia Appendix 1 Items mapped to the Measurement Instrument for Determinants of Innovations (MIDI) determinants and all questionnaire items.

10.2196/92313 Multimedia Appendix 2 Questions asked during onboarding to improve interaction with the dashboard.

10.2196/92313 Multimedia Appendix 3 Likert scores for the individual assessment and intensive care unit (ICU) overview setup.

10.2196/92313 Multimedia Appendix 4 Results: Likert scores and intention to use.

10.2196/92313 Multimedia Appendix 5 Open-ended questions and responses from the questionnaire.

10.2196/92313 Checklist 1 TIDieR checklist.

## Abbreviations

Amsterdam UMC: Amsterdam University Medical Center
FiO₂: fraction of inspired oxygen
ICU: intensive care unit
LPV: lung-protective ventilation
MIDI: Measurement Instrument for Determinants of Innovations
PEEP: positive end-expiratory pressure
TAM: technology acceptance model
TIDieR: Template for Intervention Description and Replication
V_T_/IBW: tidal volume indexed to ideal body weight
WMO: Dutch Medical Research Involving Human Subjects Act
