# Supplementary material for: Determinants of Adoption of a Mechanical Ventilation Dashboard in Intensive Care: Measurement Instrument for Determinants of Innovations–Based, Single-Center, Cross-Sectional Evaluation
Source: JMIR Hum Factors. 2026 Oct 7;13:e92313. doi: 10.2196/92313 (PMC13645201; doi:10.2196/92313)
Supplement: Supplementary file 1 [file humanfactors-v13-e92313-s001.docx]

Multimedia Appendix 1. Items mapped to Measurement Instrument for Determinants of Innovations (MIDI) determinants and all questionnaire items

| Item* | MIDI determinant | Question |
| --- | --- | --- |
| Individual assessment set-up | | |
| 1. | Procedural clarity | VentSight helps me clearly recognize when I need to adjust ventilation settings or provide additional ventilation-related care to my patient(s), based on ventilation protocols and parameters. |
| 2. | Correctness | VentSight uses data from the ventilator and threshold values from our hospital’s ventilation protocol; for me, this is the right basis to assess protocol adherence. |
| 3. | Completeness | The information VentSight displays remotely about the ventilation parameters and protocol adherence of my patient(s) is sufficiently complete to be used in clinical practice to improve ventilation care for my patient(s). |
| 4. | Complexity | VentSight is easy to understand and user-friendly, even without extensive training. |
| 5. | Compatibility | Using VentSight as a tool to remotely assess the ventilation parameters and protocol adherence of my patient(s) aligns with how we aim to work in the ICU. |
| 6. | Observability | I expect that using VentSight as a remote overview tool for my patient(s) will provide immediate insight into whether the ventilation protocol is being followed correctly. |
| 7. |  | I believe that using VentSight as a tool for remotely assessing ventilation parameters and protocol adherence will lead to: |
| 7a. | Relevance for client | better adherence to the ventilation protocol |
| 7b. | Relevance for client | shorter duration of mechanical ventilation |
| 7c. | Relevance for client | more time under lung-protective ventilation |
| 8. |  | I expect that using VentSight as a tool for remotely assessing the ventilation parameters and protocol adherence of my patient(s) will make my work: |
| 8a. | Personal benefits/ drawbacks | Easier |
| 8b. | Personal benefits/ drawbacks | More efficient |
| 8c. | Personal benefits/ drawbacks | More structured |
| 8d. | Personal benefits/ drawbacks | more tailored to individual patient care |
| 9a. | Outcome expectations | I believe it is important to keep my patient(s) within the boundaries of our ventilation protocol. |
| 9b. | Outcome expectations | I expect that using VentSight as a remote overview of my patient(s) will result in better adherence to the ventilation protocol for my patient(s). |
| 10. | Professional obligations | I consider it part of my role to use VentSight as a tool to remotely monitor the ventilation parameters and protocol adherence of my patient(s). |
| 13. | Social support | I expect that my colleagues will have a positive attitude towards using VentSight as a tool to remotely assess protocol adherence for my own patient(s). |
| 14. | Descriptive norm | I expect that I will not be the only one in the unit using VentSight, but that my colleagues will also use it. |
| 16a. | Self-efficacy | Do you think you can use VentSight to recognize when your patient(s) deviate from the ventilation protocol? |
| 16b. | Self-efficacy | Do you think you can use VentSight to determine whether your patient(s) require intervention in ventilation? |
| 16c. | Self-efficacy | Do you believe you can use VentSight to determine whether your patient(s) are deteriorating? |
| 17. | Knowledge | I believe I have sufficient knowledge about lung-protective ventilation to use VentSight effectively. |
| 18. | Awareness of content of innovation | I am familiar with the content of VentSight as a ventilation overview dashboard. |
| 23. | Time available | I think I have enough time to use VentSight to remotely assess the ventilation status and protocol adherence of my patient(s). |
| 28. | Performance feedback | I would like to receive feedback on my use of VentSight as a tool to remotely assess the ventilation protocol adherence of my own patient(s), and on the effects of this use. |
| ICU overview set-up | | |
| 1. | Procedural clarity | VentSight helps me clearly identify which patients require extra attention regarding ventilation settings, and where and when action is needed (adjusting ventilation settings) based on ventilation protocols and parameters. |
| 2. | Correctness | VentSight uses data from the ventilator and threshold values from our hospital’s ventilation protocol; for me, this is the right basis to gain a good overview and prioritise patients. |
| 3. | Completeness | The information displayed in VentSight is sufficiently complete to be useful in practice for identifying where ventilation deviates from protocol and which patients require additional ventilation-related care. |
| 4. | Complexity | VentSight is easy to understand and user-friendly, even without extensive training. |
| 5. | Compatibility | Using VentSight as a tool to gain an overview of all ventilated patients and see where ventilation deviates from protocol, so that additional care can be directed there, aligns well with how we work in the ICU. |
| 6. | Observability | I expect that using VentSight as a tool to provide an overview of all patients, and to make visible where ventilation deviates from protocol, will enable these patients to receive targeted additional attention or care. |
| 7. |  | I believe that using VentSight as an overview of ventilation parameters and protocol adherence will lead to: |
| 7a. | Relevance for client | better adherence to the ventilation protocol |
| 7b. | Relevance for client | shorter duration of mechanical ventilation |
| 7c. | Relevance for client | more time under lung-protective ventilation |
| 8. |  | I expect that VentSight, as a tool that provides an overview of the ventilation parameters and protocol adherence of all patients, will make my work: |
| 8a. | Personal benefits/ drawbacks | Easier |
| 8b. | Personal benefits/ drawbacks | More efficient |
| 8c. | Personal benefits/ drawbacks | More structured |
| 9a. | Outcome expectations | I believe it is important to keep all patients within the boundaries of our ventilation protocol. |
| 9b. | Outcome expectations | I expect that using VentSight as an overview will lead to better adherence to the ventilation protocol in patients. |
| 10. | Professional obligations | I consider it part of my role to use VentSight as a tool to monitor ventilation parameters and protocol adherence for all ventilated patients. |
| 13. | Social support | I expect that my colleagues will have a positive attitude towards using VentSight as a tool to view ventilation parameters for all patients (not just their own patients). |
| 14. | Descriptive norm | I expect that I will not be the only one unit using VentSight, but that my colleagues will also use it. |
| 16a. | Self-efficacy | Do you think VentSight can be used to identify patients who deviate from the ventilation protocol? |
| 16b. | Self-efficacy | Do you think VentSight can provide a good overview of patients’ ventilation status? |
| 16c. | Self-efficacy | Do you think that by using VentSight you can recognize which patients are deteriorating? |
| 17. | Knowledge | I believe I have sufficient knowledge about lung-protective ventilation to use VentSight effectively. |
| 18. | Awareness of content of innovation | I am familiar with the content of VentSight as a ventilation overview dashboard. |
| 23. | Time available | I believe I have enough time to use VentSight as a ventilation overview. |
| 28. | Performance feedback | I would like to receive feedback on my use of VentSight as a tool to gain an overview of all ventilated patients and protocol adherence, and on the effects of this use. |

*Question item numbers correspond to the item numbers of the matching MIDI determinants. Some item numbers are not included as these MIDI determinants were not relevant to this questionnaire.
