# Supplementary material for: Determinants of Adoption of a Mechanical Ventilation Dashboard in Intensive Care: Measurement Instrument for Determinants of Innovations–Based, Single-Center, Cross-Sectional Evaluation
Source: JMIR Hum Factors. 2026 Oct 7;13:e92313. doi: 10.2196/92313 (PMC13645201; doi:10.2196/92313)
Supplement: Supplementary file 2 [file humanfactors-v13-e92313-s002.docx]

Multimedia Appendix 2. Questions asked during onboarding to improve interaction with dashboard

| Item | Question |
| --- | --- |
| 1. | Which patients are ventilated in line with the ventilation protocol, according to the dashboard? |
| 2. | In which patients are the ventilation parameters deteriorating?? |
| 3. | In which patients are the ventilation parameters improving? |
| 4. | With what settings are these patients ventilated? |
| 5. | Which goal is set and/or reached for this patient? |
| 6. | For how long is this patient mechanically ventilated? |
| 7. | Are the criteria for weaning met in this patient? |
