# Supplementary material for: Determinants of Adoption of a Mechanical Ventilation Dashboard in Intensive Care: Measurement Instrument for Determinants of Innovations–Based, Single-Center, Cross-Sectional Evaluation
Source: JMIR Hum Factors. 2026 Oct 7;13:e92313. doi: 10.2196/92313 (PMC13645201; doi:10.2196/92313)
Supplement: Supplementary file 3 [file humanfactors-v13-e92313-s004.docx]

Multimedia Appendix 4. Results Likert scores and intention to use

|  | Response | Number (%) |
| --- | --- | --- |
| Determinants associated with the innovation | | |
| 1. Procedural clarity | Median (IQR) | 4 (4.0-4.0) |
|  | 1 | 0 |
|  | 2 | 2 (2.8%) |
|  | 3 | 7 (9.9%) |
|  | 4 | 46 (64.8%) |
|  | 5 | 13 (18.3%) |
|  | I don’t know | 3 (4.2%) |
| 2. Correctness | Median (IQR) | 4 (4.0-4.0) |
|  | 1 | 0 |
|  | 2 | 0 |
|  | 3 | 10 (14.1%) |
|  | 4 | 49 (69.0%) |
|  | 5 | 12 (16.9%) |
|  | I don’t know | 0 |
| 3. Completeness | Median (IQR) | 4 (3.2-4.0) |
|  | 1 | 1 (1.4%) |
|  | 2 | 2 (2.8%) |
|  | 3 | 14 (19.7%) |
|  | 4 | 38 (53.5%) |
|  | 5 | 11 (15.5%) |
|  | I don’t know | 5 (7.0%) |
| 4. Complexity | Median (IQR) | 4 (4.0-5.0) |
|  | 1 | 1 (1.4%) |
|  | 2 | 2 (2.8%) |
|  | 3 | 6 (8.5%) |
|  | 4 | 40 (56.3%) |
|  | 5 | 19 (26.8%) |
|  | I don’t know | 3 (4.2%) |
| 5. Compatibility | Median (IQR) | 4 (3.5-4.0) |
|  | 1 | 1 (1.4%) |
|  | 2 | 1 (1.4%) |
|  | 3 | 15 (21.1%) |
|  | 4 | 36 (50.7%) |
|  | 5 | 14 (19.7%) |
|  | I don’t know | 4 (5.6%) |
| 6. Observability | Median (IQR) | 4 (4.0-4.0) |
|  | 1 | 1 (1.4%) |
|  | 2 | 1 (1.4%) |
|  | 3 | 8 (11.3%) |
|  | 4 | 42 (59.2%) |
|  | 5 | 16 (22.5%) |
|  | I don’t know | 3 (4.2%) |
| 7. Relevance for client (a) | Median (IQR) | 4 (3.0-4.0) |
|  | 1 | 1 (1.4%) |
|  | 2 | 5 (7.0%) |
|  | 3 | 17 (23.9%) |
|  | 4 | 32 (45.1%) |
|  | 5 | 13 (18.3%) |
|  | I don’t know | 3 (4.2%) |
| 8. Relevance for client (b) | Median (IQR) | 3 (2.0-3.0) |
|  | 1 | 1 (1.4%) |
|  | 2 | 16 (22.5%) |
|  | 3 | 29 (40.8%) |
|  | 4 | 15 (21.1%) |
|  | 5 | 0 |
|  | I don’t know | 10 (14.1%) |
| 9. Relevance for client (c) | Median (IQR) | 4 (4.0-4.0) |
|  | 1 | 1 (1.4%) |
|  | 2 | 3 (4.2%) |
|  | 3 | 11 (15.5%) |
|  | 4 | 46 (64.8%) |
|  | 5 | 6 (8.5%) |
|  | I don’t know | 4 (5.6%) |
| Determinants associated with the adopting user | | |
| 10. Personal benefits/ drawbacks (a) | Median (IQR) | 4 (3.0-4.0) |
|  | 1 | 1 (1.4%) |
|  | 2 | 4 (5.6%) |
|  | 3 | 18 (25.4%) |
|  | 4 | 35 (49.3%) |
|  | 5 | 8 (11.3%) |
|  | I don’t know | 5 (7.0%) |
| 11. Personal benefits/ drawbacks (b) | Median (IQR) | 4 (3.5-4.0) |
|  | 1 | 1 (1.4%) |
|  | 2 | 3 (4.2%) |
|  | 3 | 13 (18.3%) |
|  | 4 | 43 (60.6%) |
|  | 5 | 7 (9.9%) |
|  | I don’t know | 4 (5.6%) |
| 12. Personal benefits/ drawbacks (c) | Median (IQR) | 4 (3.0-4.0) |
|  | 1 | 1 (1.4%) |
|  | 2 | 5 (7.0%) |
|  | 3 | 16 (22.5%) |
|  | 4 | 42 (59.2%) |
|  | 5 | 5 (7.0%) |
|  | I don’t know | 2 (2.8%) |
| 13. Outcome expectations (a) | Median (IQR) | 5 (4.0-5.0) |
|  | 1 | 1 (1.4%) |
|  | 2 | 0 |
|  | 3 | 1 (1.4%) |
|  | 4 | 32 (45.1%) |
|  | 5 | 36 (50.7%) |
|  | I don’t know | 1 (1.4%) |
| 14. Outcome expectations (b) | Median (IQR) | 4 (3.5-4.0) |
|  | 1 | 2 (2.8%) |
|  | 2 | 0 |
|  | 3 | 15 (21.1%) |
|  | 4 | 43 (60.6%) |
|  | 5 | 7 (9.9%) |
|  | I don’t know | 4 (5.6%) |
| 15. Professional obligation | Median (IQR) | 3.5 (3.0-4.0) |
|  | 1 | 4 (5.6%) |
|  | 2 | 11 (15.5%) |
|  | 3 | 17 (23.9%) |
|  | 4 | 26 (36.6%) |
|  | 5 | 6 (8.5%) |
|  | I don’t know | 7 (9.9%) |
| 16. Social support | Median (IQR) | 4 (3.0-4.0) |
|  | 1 | 2 (2.8%) |
|  | 2 | 5 (7.0%) |
|  | 3 | 20 (28.2%) |
|  | 4 | 36 (50.7%) |
|  | 5 | 2 (2.8%) |
|  | I don’t know | 6 (8.5%) |
| 17. Descriptive norm | Median (IQR) | 4 (3.0-4.0) |
|  | 1 | 1 (1.4%) |
|  | 2 | 4 (5.6%) |
|  | 3 | 14 (19.7%) |
|  | 4 | 39 (54.9%) |
|  | 5 | 7 (9.9%) |
|  | I don’t know | 6 (8.5%) |
| 18. Self-efficacy (a) | Median (IQR) | 4 (4.0-4.0) |
|  | 1 | 1 (1.4%) |
|  | 2 | 2 (2.8%) |
|  | 3 | 6 (8.5%) |
|  | 4 | 44 (62.0%) |
|  | 5 | 17 (23.9%) |
|  | I don’t know | 1 (1.4%) |
| 19. Self-efficacy (b) | Median (IQR) | 4 (4.0-4.0) |
|  | 1 | 1 (1.4%) |
|  | 2 | 2 (2.8%) |
|  | 3 | 13 (18.3%) |
|  | 4 | 41 (57.7%) |
|  | 5 | 11 (15.5%) |
|  | I don’t know | 3 (4.2%) |
| 20. Self-efficacy (c) | Median (IQR) | 4 (4.0-4.0) |
|  | 1 | 1 (1.4%) |
|  | 2 | 2 (2.8%) |
|  | 3 | 12 (16.9%) |
|  | 4 | 46 (64.8%) |
|  | 5 | 9 (12.7%) |
|  | I don’t know | 1 (1.4%) |
| 21. Knowledge | Median (IQR) | 4 (3.0-4.0) |
|  | 1 | 3 (4.20%) |
|  | 2 | 6 (8.5%) |
|  | 3 | 15 (21.1%) |
|  | 4 | 31 (43.7%) |
|  | 5 | 15 (21.1%) |
|  | I don’t know | 1 (1.4%) |
| 22. Awareness of content of innovation | Median (IQR) | 4 (3.0-4.0) |
|  | 1 | 5 (7.0%) |
|  | 2 | 8 (11.3%) |
|  | 3 | 15 (21.1%) |
|  | 4 | 34 (47.9%) |
|  | 5 | 6 (8.5%) |
|  | I don’t know | 3 (4.2%) |
| Determinants associated with the organisation | | |
| 23. Time available | Median (IQR) | 4 (3.0-4.0) |
|  | 1 | 2 (2.8%) |
|  | 2 | 5 (7.0%) |
|  | 3 | 23 (32.4%) |
|  | 4 | 32 (45.1%) |
|  | 5 | 3 (4.2%) |
|  | I don’t know | 6 (8.5%) |
| 24. Performance feedback | Median (IQR) | 4 (3.0-4.0) |
|  | 1 | 3 (4.2%) |
|  | 2 | 8 (11.3%) |
|  | 3 | 15 (21.1%) |
|  | 4 | 36 (50.7%) |
|  | 5 | 6 (8.5%) |
|  | I don’t know | 3 (4.2%) |
| Intended use of the dashboard |  |  |
|  | Yes | 51 (71.8%) |
|  | No | 0 |
|  | Maybe | 20 (28.2%) |
