# Supplementary material for: Determinants of Adoption of a Mechanical Ventilation Dashboard in Intensive Care: Measurement Instrument for Determinants of Innovations–Based, Single-Center, Cross-Sectional Evaluation
Source: JMIR Hum Factors. 2026 Oct 7;13:e92313. doi: 10.2196/92313 (PMC13645201; doi:10.2196/92313)
Supplement: Supplementary file 4 [file humanfactors-v13-e92313-s005.docx]

Multimedia Appendix 5. Open ended questions and responses from the questionnaire

| **No.** | **Role** | | | **Would you like to use VentSight in your work?** | **Why would you not want to use VentSight?** | |
| --- | --- | --- | --- | --- | --- | --- |
| 1 | ICU nurse >5 yrs | | | Yes |  | |
| 2 | ICU nurse <5 yrs | | | Maybe |  | |
| 3 | ICU nurse >5 yrs, ventilation | | | Yes | No | |
| 4 | ICU nurse ie** | | | Yes |  | |
| 5 | ICU nurse <5 yrs | | | Maybe | Too much work/time | |
| 6 | ICU nurse >5 yrs, ventilation practitioner | | | Yes |  | |
| 7 | ICU Nurse <5 yrs | | | Yes |  | |
| 8 | ICU nurse <5 yrs | | | Yes |  | |
| 9 | ICU nurse <5 yrs | | | Yes |  | |
| 10 | ICU nurse <5 yrs | | | Yes |  | |
| 11 | ICU nurse >5 yrs | | | Yes |  | |
| 12 | ICU nurse <5 yrs | | | Yes |  | |
| 13 | ICU nurse <5 yrs | | | Yes |  | |
| 14 | ICU nurse >5 yrs | | | Maybe | I would like the dashboard to be available on the bedside PCs so it can be used during ward rounds. | |
| 15 | ICU nurse ie** | | | Maybe | I am in training and well informed about lung-protective ventilation and asynchrony—so personally, I do not find it necessary. Perhaps just to occasionally check trends. As long as alarm limits are set tightly, you will be alerted in time. | |
| 16 | ICU nurse <5 yrs | | | Yes |  | |
| 17 | ICU nurse >5 yrs | | | Yes |  | |
| 18 | ICU nurse ie**, MC^#^ nurse | | | Yes |  | |
| 19 | ICU nurse >5 yrs | | | Yes |  | |
| 20 | ICU nurse <5 yrs | | | Yes |  | |
| 21 | ICU nurse >5 yrs | | | Maybe |  | |
| 22 | ICU nurse >5 yrs | | | Yes |  | |
| 23 | ICU nurse >5 yrs | | | Yes |  | |
| 24 | ICU nurse <5 yrs | | | Yes |  | |
| 25 | ICU nurse <5 yrs | | | Maybe | Because it would be an extra step, with information that may already be available in familiar systems. | |
| 26 | ICU nurse >5 yrs | | | Yes |  | |
| 27 | ICU nurse <5 yrs | | | Yes |  | |
| 28 | ICU nurse ie** | | | Yes |  | |
| 29 | ICU nurse <5 yrs | | | Maybe | It should not become a tool for monitoring or policing each other—purely for analysis and education. | |
| 30 | ICU nurse >5 yrs | | | Yes |  | |
| 31 | ICU nurse >5 yrs | | | Yes |  | |
| 32 | Resident | | | Yes |  | |
| 33 | Intensivist | | | Yes |  | |
| 34 | Intensivist | | | Yes |  | |
| 35 | Resident | | | Yes |  | |
| 36 | Researcher^$^ | | | Yes |  | |
| 37 | Researcher^$^ | | | Yes |  | |
| 38 | ICU nurse >5 yrs, VP* | | | Yes |  | |
| 39 | Intensivist | | | Maybe | Another extra screen. | |
| 40 | Researcher^$^ | | | Maybe | It provides a beautiful overview, but not much new information. The questions are mainly about whether it is a good dashboard, and yes, I think it is. But this information can also be obtained in other ways via the existing infrastructure. | |
| 41 | Intensivist | | | Yes |  | |
| 42 | Medical intern | | | Yes |  | |
| 43 | Medical intern | | | Maybe | I wonder what it really adds beyond the tab in Epic. Moreover, I already find Epic a complicated program with many ways to view/find the same information. This would just be yet another way to access the same data. | |
| 44 | Resident | | | Yes |  | |
| 45 | Intensivist | | | Yes |  | |
| 46 | Medical intern | | | Yes |  | |
| 47 | Resident | | | Maybe | I think an extra screen could hinder daily use, similar to the overview of hemodynamic parameters. | |
| 48 | Intensivist | | | Maybe |  | |
| 49 | Resident | | | Yes |  | |
| 50 | Researcher^$^ | | | Maybe | As a researcher, it is less relevant, but as a physician, I would like to use it. | |
| 51 | Intensivist | | | Yes |  | |
| 52 | Resident | | | Yes |  | |
| 53 | ICU nurse >5 yrs, VP | | | Yes | Quick overview and less time needed to screen all patients. | |
| 54 | resident | | | Yes |  | |
| 55 | ICU nurse >5 yrs, VP | | | Yes |  | |
| **No.** | | **Why would you want to use VentSight?** | | | | **What do you see as potential advantages of using VentSight in your work?** |
| 1 | | Provides a quick overview of the entire unit. Also useful during meetings. | | | |  |
| 2 | |  | | | | Ability to monitor multiple patients from a distance. |
| 3 | | Ability to gain insights from multiple locations and see at a glance how ventilation is going. | | | |  |
| 4 | | Overview of ventilation parameters, including curves. This way, you can continuously monitor how the patient is doing. | | | | I find VentSight more structured than the Hamilton itself and I can see more at a glance. |
| 5 | |  | | | | Insight into difficult-to-ventilate patients. |
| 6 | | Overview of all patients to identify those needing extra attention. | | | | Overview of patients when doing ward rounds as a working group |
| 7 | | Convenient overview. | | | | Quick insight into patients’ ventilation status. Protocol nearby. |
| 8 | | More insight into lung-protective ventilation and threshold monitoring. Also insight into whether someone is ready to be weaned. | | | | Clear overview, better patient care. |
| 9 | | Looks well-organized. | | | | Faster insight into whether lung-protective ventilation is being applied |
| 10 | | Helps optimize ventilation and lung-protective ventilation. Also helps with weaning. | | | | Faster insight into trends, values, and monitoring. |
| 11 | | I think it can be useful during discussion moments such as ward rounds. I just think it doesn’t have much added value beyond that. | | | | Long-term overview. |
| 12 | | Clear | | | | Overview and measurement parameters. |
| 13 | | Clear and well-structured. | | | | In een oogopslag duidelijk overzicht en mogelijke vervanderingen |
| 14 | |  | | | | Clear overview at a glance and possible changes. |
| 15 | |  | | | | Problems are detected earlier. |
| 16 | | As a supplement to clinical reasoning with trainees. More structured than via EPIC and the Hamilton ventilator monitor. | | | | More structured overview, making clinical reasoning easier. |
| 17 | | Good way to provide training to students. | | | |  |
| 18 | | I think it is useful and clear, and sometimes also nice to be able to view ventilation remotely. | | | | Viewing ventilation remotely, long-term overview, clear display of adjustments made. |
| 19 | | Clear, easy to use. | | | | Quick ventilation overview. |
| 20 | | I think it’s a nice extra tool, but not strictly necessary to ventilate optimally, since all the data is also available on the ventilator. I think it is mainly an addition for reviewing adjustments and why they may have been made, since this is currently poorly documented. | | | |  |
| 21 | |  | | | | Trend monitoring. |
| 22 | | Neat overview that provides simple, clear information. | | | | Protocol adherence may result in shorter ventilation duration. |
| 23 | | Clear overview and a tool to potentially adjust ventilation settings. | | | | Consistent view regarding ventilation. |
| 24 | | Better overview of ventilation in the past hours. | | | | Quicker clear overview instead of searching back in EPIC. |
| 25 | |  | | | | A clear overview with key highlights. |
| 26 | | Convenient, insightful, clear. | | | |  |
| 27 | | Good for use in clinical reasoning about ventilation. Keeps you alert to values that may need to be adjusted. | | | | Creating more connection with ventilation. |
| 28 | | Trend monitoring is more structured. | | | |  |
| 29 | |  | | | | Analysis and training. |
| 30 | | Overview, easy to combine with protocol. | | | | Direct overview. |
| 31 | |  | | | |  |
| 32 | | Easy to use. | | | | Overview. |
| 33 | |  | | | | Better insight into lung protectiveness and success of weaning. |
| 34 | | Clear and simple overview, with the most important parameters visible at a glance. | | | | Quick overview, useful for supervisors to prioritize ward rounds, for example. |
| 35 | | Good overview, pleasant to use during my shifts. | | | | Then I don’t have to click on all my patients in EPIC. |
| 36 | | As a researcher, I think it’s an insightful way to see which patients are being ventilated and for how long.. | | | | From the ventilation duration, I can see which patients are eligible for the study. It can also help estimate how long a patient will remain ventilated. |
| 37 | | Nice overview. | | | | More focus on ventilation quality. |
| 38 | | Provides an overview of ventilation settings and any deviations. | | | | Increased awareness of settings. |
| 39 | |  | | | | Better overview over time. |
| 40 | |  | | | | With EPIC overview lists, you could also add goals that force doctors/nurses to think about ventilation targets, hopefully leading to better ventilation |
| 41 | | Which patients are not ventilated according to protocol. | | | | Faster identification of which patient is being ventilated lung-protectively. |
| 42 | | Education for medical students | | | | Understanding ventilation. |
| 43 | |  | | | | Overview at the nursing station of all patients’ ventilation settings. |
| 44 | | Clear and easy to use. | | | | Clear overview. |
| 45 | | As a tool for myself. | | | | Hopefully better ventilation for my patients. |
| 46 | | As a tool for myself. | | | | Better bird’s-eye view of all patients in the unit. |
| 47 | |  | | | | Easy overview and correlation with clinic |
| 48 | |  | | | | Mainly insight into what has happened in the past 24 hours, not so much what is wrong at the moment—because we are at the bedside for that. |
| 49 | | Clear real-time overview of the entire unit at a glance, especially useful for evening/night shifts. | | | | Time savings during, for example, night shifts, and the ability to prioritize sicker patients more quickly through a quick overview. |
| 50 | |  | | | | Easy to get an overview. |
| 51 | | Easier trend overview compared to Epic. | | | |  |
| 52 | |  | | | | Clear overview of abnormal values. |
| 53 | |  | | | | Faster patient prioritization. Ability to assist remotely through more insight into ventilation data. |
| 54 | | Clear and intuitive dashboard. I personally have some experience with ventilation, but not with EPIC. Therefore, I think this dashboard could go live immediately. | | | | Complete and especially fast overview of all ventilated patients in your unit. Also very useful during shifts. |
| 55 | | I think it adds value for members of the ventilation working group. The aim is to conduct ventilation rounds, but this is often not possible because you have to physically walk through all units, meaning you are away from your own unit. This also takes a lot of time. Now you can simply check on your own unit where potential problems are and then go directly to that patient. You can also provide more explanation to the nurse at the bedside. | | | | time savings — more training for nurses — more frequent ability to conduct ventilation rounds, even during less staffed shifts like night shifts. |
| **No.** | | | **What do you see as potential disadvantages of using VentSight in your work?** | | | **Do you have any suggestions?** |
| 1 | | | None | | |  |
| 2 | | |  | | |  |
| 3 | | |  | | |  |
| 4 | | | Looking more at the screens instead of at the patient. | | | Make VentSight easily available on the bedside computers so that the patient’s condition can also be assessed when reviewing ventilation parameters. |
| 5 | | | Not looking at the patient anymore. | | |  |
| 6 | | |  | | |  |
| 7 | | | Extra screen, initial adjustment period needed. Must also be displayed on the screen at the patient’s bedside. | | |  |
| 8 | | |  | | |  |
| 9 | | | I can’t think of any disadvantages right now | | | A general ventilation screen on the unit, with the possibility to dive deeper into trends at the patient’s bedside. |
| 10 | | | Less attention to the patient, more focus on numbers. | | |  |
| 11 | | | Many parameters, which may create noise. | | |  |
| 12 | | | Another device with data. | | | A computer at the nurses’ station that is always on, so you don’t have to perform extra actions to get the overview. |
| 13 | | |  | | |  |
| 14 | | | The extra step of opening the dashboard. | | |  |
| 15 | | | More searching and interest needed in how it works. | | | I would prefer it on the ventilator monitor. |
| 16 | | | I work at another location as well with the SERVO, and this program is not supported there. VentSight is not complete because it lacks the clinical impression of the patient. All the data is essentially already in EPIC and the Hamilton monitor, so it does not provide new insights—only the layout is much more structured. | | |  |
| 17 | | |  | | |  |
| 18 | | | That it may not be used, or not used enough. | | |  |
| 19 | | | It might make people lazy. Everyone might get involved. | | |  |
| 20 | | | I’m afraid nurses will use it little, especially now that it cannot yet be opened in patient rooms. I wonder if it truly adds value… I also think doctors will use it more than nurses. | | |  |
| 21 | | | If it is only available at the nurses’ station, I think few people will use it. So please also make it available on the bedside computers. | | |  |
| 22 | | | Possible interference from colleagues. | | |  |
| 23 | | | None | | |  |
| 24 | | |  | | |  |
| 25 | | | Too much extra information, possibly unreliable information. | | | Start a pilot so that we can work with it and form a better opinion. |
| 26 | | |  | | |  |
| 27 | | | That colleagues will look more at numbers and less at the patient. Less thinking for themselves. | | | None |
| 28 | | | Extra thing to monitor. | | |  |
| 29 | | | ‘People might get lazier,’ less bedside teaching and more focus on numbers. | | |  |
| 30 | | | As a nurse, you are always at the bedside, directly next to the ventilator. I think it is especially a nice tool for doctors to use remotely. | | |  |
| 31 | | |  | | | Ensure that you don’t have to log in individually, but that it is continuously visible on a screen, just like monitoring and infusion pump surveillance. |
| 32 | | | Less contact and overview of the patient. | | |  |
| 33 | | |  | | |  |
| 34 | | |  | | | Ideally, such a dashboard would be integrated into EPIC instead of using another system with a separate login, etc. |
| 35 | | | Separate program from EPIC. Working in different programs is inconvenient. | | |  |
| 36 | | | The only thing I can think of is (yet) another tab to open. | | | For what I would use it for, I have no suggestions. |
| 37 | | | None | | |  |
| 38 | | | That more attention is paid to settings rather than the clinical situation. | | |  |
| 39 | | |  | | |  |
| 40 | | | It is limited to ventilation data. It is again new information on a new platform. It only adds goals and an overview—why can’t this be in EPIC? EPIC also has reports that can provide an overview. Also, you only have ventilation data now. It would be insightful if you could also add blood gas analyses to support certain ventilation goals. Lastly, sedation is also an important parameter for starting weaning. This is missing. | | | Good initiative, but use systems we already have (EPIC report overview). Add blood gas and sedation. |
| 41 | | | None | | | None |
| 42 | | | Less bedside presence. | | | Include an educational component! |
| 43 | | | Extra hassle, see question 1. | | | Add color to make it more visually clear. |
| 44 | | | Too much information | | |  |
| 45 | | | It gives a bit of a “big brother is watching you” feeling. So it’s important not to target individuals or make “ranking lists” of who ventilates the best. | | |  |
| 46 | | | Less patient contact. | | | none |
| 47 | | | Extra screen. | | |  |
| 48 | | |  | | |  |
| 49 | | | Possibly too many “skippers” (too frequent changes/fluctuations in policy). | | | Regarding the question of whether protocol adherence would actually improve or lead to shorter ventilation duration, in my opinion, research should determine this—you cannot know this now. Especially regarding ventilation duration, I have doubts, as it would also have to mean that we are certain the current protocols are the most effective. Furthermore, I think the dashboard could be very useful for education. |
| 50 | | |  | | | Perhaps also add driving pressure and respiratory rate to the first overview screen with all patients. |
| 51 | | |  | | |  |
| 52 | | | Maybe looking less at the patient, not directly seeing which medication is on the pumps, no view of further hemodynamics (because you’re not at the patient). | | |  |
| 53 | | |  | | |  |
| 54 | | | An extra screen. | | | A browser pop-up for preselected patients would be useful to me. This way, the overview does not fade into the background but instead prompts attention. |
| 55 | | |  | | |  |

*Ventilation Practitioner; ** ICU nurse in education, ^#^Medium Care nurse, ^$^Researcher in the field of mechanical ventilation
